# Femtosecond Laser-Engineered Sustainable Glass Surfaces with Tunable Wettability Properties for Photovoltaic System Applications

**DOI:** 10.3390/nano16080475

**Published:** 2026-04-17

**Authors:** Emil Filipov, Liliya Angelova, Aleksandra Zhelyazkova, Albena Daskalova

**Affiliations:** Institute of Electronics, Bulgarian Academy of Sciences, 1784 Sofia, Bulgaria; liliyaangelova9@gmail.com (L.A.); alexandra_jivkova@abv.bg (A.Z.)

**Keywords:** femtosecond laser structuring, photovoltaic panels, superhydrophilic surfaces

## Abstract

This study investigates the femtosecond laser surface texturing approach to tune the wetting properties of glass substrates applied for photovoltaic panels. Two types of microstructured LIPSS-containing motifs—parallel channels and intersecting (crossing) patterns—were fabricated and evaluated through comprehensive durability tests, including thermal cycling, UV exposure, chemical immersion, mechanical abrasion, and dust retention assessment. Wettability measurements showed that both textures exhibit stable hydrophilicity behavior, with the intersecting patterns exhibiting the fastest wetting dynamics; in many cases, complete surface wetting occurred within the first few minutes, preventing a measurable contact angle at later stages. The durability tests caused only minor smoothing of the textured features, and the overall micro- and nanostructures remained intact. Optical characterization revealed that the laser-induced textures maintained high transmittance with no significant degradation after environmental exposure. Overall, the results demonstrate that femtosecond laser texturing provides a robust, coating-free method for producing stable and tunable wetting behavior on glass, offering a promising pathway for the future creation of durable, highly hydrophilic self-cleaning surfaces in photovoltaic systems.

## 1. Introduction

The rapid increase in global energy demand and the expected depletion of current “gold standard” fossil fuels have created the urgency of transitioning toward renewable and cleaner energy sources [1]. Solar power has been established as one of the most promising candidates for sustainable energy generation for its clean high energy, safety and lack of residual pollution. Photovoltaic (PV) technologies have achieved remarkable advances in efficiency and scalability due to their relatively lower manufacturing and market price as well as their low maintenance cost [1,2]. PV technology relies on the photovoltaic effect, which is based on the transformation of light energy into electrical energy by inducing directional current flow within a semiconductor material. Standard PVs are composed of a semiconductor core (e.g., crystalline silicon) that is encapsulated in polymeric and antireflecting glass covers which ensure the minimum loss of solar energy upon contact with the panel [3,4].

Despite the high efficiency of PVs, practical limitations related to surface contamination remain significant obstacles to their long-term performance [5]. Dust, organic residues, and moisture accumulation on solar panel surfaces can reduce light transmission, thereby lowering their overall efficiency. Soiling requires the mechanical cleaning of the panels (blowing, brushing), which results in increased maintenance costs and could potentially lead to unwanted damage to the PV surface, compromising their conversion efficiency [1]. Approaches to overcoming this limitation include techniques that repel electrostatic interactions, as well as the engineering of surface coatings or topographies that enable self-cleaning properties through either superhydrophobicity or superhydrophilicity. The creation of superhydrophobic surfaces has been inspired by the natural properties of lotus leaves, which are decorated with hierarchical micro- and nano-topographical features that facilitate the sliding of falling water droplets and do not allow them to remain spread out on the surface [5,6]. Their “cleaning” properties arise from the fact that water droplets lose their elastic properties, and as they slide off the superhydrophobic surface, they can stick to and remove accumulated particles [5]. On the other hand, superhydrophilicity is another physicochemical phenomenon that could enhance the self-cleaning properties of a surface. In this case, water forms a continuous stream rather than remaining in a droplet shape, and hence, it can wash away debris [7].

Different techniques for the development of both types of structures, based on surface modifications, have been explored in order to enhance topography and roughness. These include photolithography, plasma-based spraying, chemical deposition, and sol–gel methods [5]. The key disadvantages of these techniques include the need for complex equipment and additional materials (i.e., chemical precursors), environmental concerns, and the risk of the delamination of deposited layers. A potent alternative to these techniques is the femtosecond (fs) laser texturing of surfaces, which allows for very precise localized modification at both the micro- and nanoscales [8]. Due to the combination of extremely high pulse energy, leading to material evaporation without melting, and a very short pulse duration, the fs laser processing of surfaces leads to the generation of well-defined micro- and nano-patterns without the unspecific thermal damage of surfaces [8,9]. By controlling the laser working parameters (laser fluence, pulse repetition rate, and scanning velocity), the morphological features of the created patterns, such as dimensions, depth, and spacing, can be precisely controlled [10]. In the research of Lin et al., the fs laser ablation of silica glass with a varying number of applied pulses led to the formation of aligned micro-pits with submicron ripples [11]. The patterns significantly increased the level of hydrophobicity of the samples, making them superhydrophobic. Furthermore, this study’s results indicated that the presence of hierarchical micro- and nano-features within the laser-induced pits improved the anti-scattering properties and incident light transmittance of the surfaces, compared to the control ones. In a paper by Deng and Ki, it was revealed how fs laser radiation could create microvoid arrays inside a glass substrate [12]. The change in the separation distance between microvoids was crucial for the wettability of processed surfaces. The smaller distance between 10 and 15 μm ensured a superhydrophilic medium with a contact angle smaller than 5°.

In this context, fs laser surface processing has emerged as a promising, eco-friendly, green technique for tailoring surface wettability without the use of chemical coatings or mechanical treatments. Laser-induced surface structures can exert control over wettability, enabling either superhydrophobic or superhydrophilic behavior while preserving the intrinsic optical transparency and photovoltaic performance of the underlying material [13].

Femtosecond laser processing is known to induce either superhydrophilic or superhydrophobic behavior on glass surfaces, depending strongly on the applied processing conditions. This apparent duality arises from the interplay between surface morphology, wetting regime, and surface chemistry. From a topographical perspective, laser-induced roughness can promote either Wenzel-type wetting, where liquid fully penetrates surface asperities and enhances intrinsic wettability, or Cassie–Baxter wetting, where trapped air pockets reduce solid–liquid contact and increase apparent hydrophobicity [13]. The transition between these regimes is strongly influenced by laser parameters such as fluence, pulse overlap, scanning speed, and the number of processing passes, which determine the resulting feature size, depth, and hierarchical organization [14]. Typically, the formation of well-defined laser-induced periodic surface structures (LIPSSs) combined with moderate microscale roughness promotes capillary-driven spreading and hydrophilicity. In contrast, more complex multiscale roughness with pronounced microcavities can stabilize air trapping and lead to superhydrophobic behavior [15,16]. In addition to morphology, femtosecond laser irradiation induces significant changes in surface chemistry. The generation of surface defects, increased oxygen content, and hydroxyl group formation enhances surface energy and contributes to the initially observed superhydrophilic behavior. However, post-processing environmental exposure plays a critical role: the adsorption of airborne hydrocarbons can progressively reduce surface energy and induce a transition toward hydrophobicity, a phenomenon often referred to as hydrophobic recovery [8,17]. In the present study, laser processing parameters were selected to promote LIPSS-dominated hierarchical structures with controlled roughness and high surface energy, thereby favoring stable hydrophilic behavior. This approach is particularly relevant for photovoltaic applications, where uniform water spreading is advantageous for anti-soiling functionality.

The goal of the present study was to propose a novel experimental methodology for addressing key surface-related limitations in PV systems by the application of fs laser texturing to create highly hydrophilic self-cleaning surfaces. For photovoltaic applications, superhydrophilic surfaces may offer advantages over superhydrophobic ones, as they promote uniform water film formation rather than “lotus leaf” droplet rolling. This ensures a more homogeneous removal of fine dust particles and reduces optical scattering from residual droplets, which is critical for maintaining optical efficiency. Since the frontsheet of conventional PV systems that is in contact with the environment is based on glass, soda–lime glass was the material chosen for the purpose of this experimental study. The surfaces of soda–lime glass samples were treated with a femtosecond (fs) laser in order to create superficial micro- and nanoscaled patterns with pre-defined varying morphologies. The validation of this experimental work involved the completion of a series of durability tests with both control and fs laser-processed samples, whose aim was to simulate various environmental conditions that could possibly affect PVs’ performance and efficiency of energy conversion in real life. Particular properties and features of the materials relevant to their applications in solar panels, such as wettability, reflectance, transmittance, morphology, and surface roughness, were compared before and after the execution of the durability tests to compare whether the fs laser-induced topographic patterns withstand and endure regarding morphology and surface-related properties.

## 2. Materials and Methods

### 2.1. Surface Processing of Soda–Lime Silicate Glass by fs Laser Irradiation

**Figure 1:**
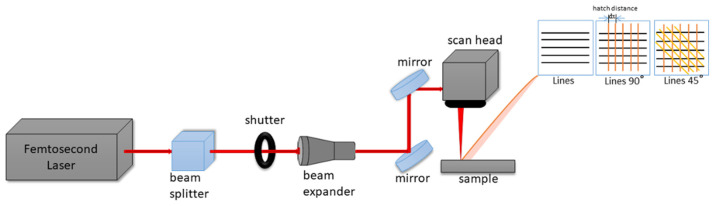
A scheme of the fs laser sample system equipped with SCANLAB’s IntelliSCAN III 14 used for creating patterns intersecting at a varying degree. An example of the created patterns is depicted in this figure, where black lines represent the first layer, orange lines (at 90° with respect to black lines) the second layer, and yellow lines (at 45°; with respect to orange lines) the third layer.

The surfaces of soda–lime glass samples (DIN ISO 8037-1) [18] were ablated with a Ti:sapphire femtosecond laser system (Solstice Ace, MKS Spectra Physics, CA, USA), operating with a central wavelength (λ) of 800 nm and a pulse width (τ) of 70 fs. The repetition rate of laser radiation (υ) was set at 1 kHz. After the initial optimization of the primary experimental procedure involving the laser processing of samples both in and out of focus, with varying laser power (P = 20–60 mW) and scanning velocity (v = 1–32 mm/s), it was determined that only surfaces bearing laser-induced periodic surface structures (LIPSSs) will be involved in further experimentation due to their ability to shift surface wettability towards superhydrophilicity. The formation of LIPSSs was achieved after the laser processing of the glass surface out of focus, P = 40 mW, v = 1 mm/s. In order to obtain reproducible and highly precise surface patterns with defined morphologies, a galvanometer scanner, SCANLAB’s IntelliSCAN III 14 system (Scanlab, Puchheim, Germany), was used. All samples were positioned on a stage under the scanning system perpendicularly to the incident laser beam, as the morphological designs together with scanning parameters, such as hatch distance (d_x_), velocity (v), the number of intersecting layers and the angle of subsequent layers, were pre-defined using LaserDesk 1.6.23.0 software. A scheme of the laser system and the design of sample processing is presented in Figure 1. The materials were processed using working parameters: P = 40 mW; v = 1 mm/s; number of intersecting layers: 1–3; intersecting angle: 45°–90°.

### 2.2. Characterization of Laser-Induced Surface Morphology

Initial surface characterization was performed using scanning electron microscopy (SEM) (“Lyra”, Tescan Orsay Holding, Brno-Kohoutovice, Czech Republic). All samples were sputter-coated with a nanometric layer of carbon before analysis. Images were obtained at 20 kV. The SEM system further allowed for energy-dispersive X-ray spectroscopy (EDX) with its additional module (Quantax 200, Bruker, Karlsruhe, Germany). A separate morphological analysis based on optical profilometry was carried out. An optical profilometer (Zeta-20 with a Zeta Optics Module, Zeta Instruments, Gallatin Valley, MT, USA) provided clear insight into the spatial features of the laser-induced patterns as it was possible to obtain 3D true-color images of the surfaces. All images were acquired at a magnification of 20× with a field of view of 476 × 357 μm. To measure surface roughness, the profiles of the true-color images were analyzed, and the Sa (arithmetic mean height, ISO 25178 [19]) parameter was taken into account [20]. Sa values represent the average of five independent measurements, carried out on both laser-modified and untreated regions of the samples. The measurements were performed using the integrated software provided by the manufacturer (Zeta Instruments, Milpitas, CA, USA).

### 2.3. Assessment of Surface Wettability

Surface wettability measurements were carried out by monitoring the change in the contact angle of a droplet of distilled water (2 μL), deployed on the surface of laser-treated and control samples. For this purpose, DSA100 Drop Shape Analyzer (KRUSS GmbH, Hamburg, Germany) was used. By using ADVANCE software Version 1.14 (KRUSS GmbH, Hamburg, Germany), the evolution of the drop’s contact angle was followed for 180 s as measurements were taken at each second during the first minute and then at each minute for the remaining time/120 s or two minutes. For each laser-structured glass specimen, measurements were taken at three separate positions on five different samples; the results were averaged and referred to those taken from the control surface.

### 2.4. Measuring the Transmittance of Soda–Lime Glass Samples

The optical properties of glass are of key importance for its application in solar panels in order to maintain their highest efficiency [21]. Glass surfaces were first irradiated with an fs laser in the above-mentioned manner, and subsequently, their optical properties were evaluated. These were further analyzed and compared before and after the series of durability tests that will be discussed below. The transmittance of the samples was monitored by using an Optical Transmittance Spectrometer (Ocean Optics Inc., Orlando, FL, USA). The OTS is built on a high-resolution miniature linear CCD array spectrometer configured for the 380–780 nm wavelength range. The system ensures a transmittance accuracy to ±1.0% and precision to ±0.1%.

### 2.5. Durability Tests for Simulation of Real-Life Conditions on Control and fs Laser-Processed Soda–Lime Glass

All the durability tests were carried out using samples of soda–lime glass (DIN ISO 8037-1). For the initial parametric tests, the following working parameters of the fs laser irradiation conditions were chosen for the soda–lime glass samples: P = 40 mW; v = 1 mm/s; d_x_ = 25 µm; number of overlying patterns either 0 (parallel lines) or 3, crossing at 60° with respect to the prior pattern.

For each group of samples, a respective control sample was included in all tests. For each test, new samples bearing the respective fs laser-induced modifications were prepared. Separately, samples processed with the same laser parameters were subjected to all durability tests in order to evaluate the behavior of the surface patterns after all tests and how they would affect the physicochemical and optical properties of the samples. The morphology and optical properties of the samples were analyzed and compared after each test. For combined durability testing, samples were subjected sequentially to thermal cycling, UV exposure, chemical treatment, abrasion, and dust exposure in this order.

#### 2.5.1. Thermal Treatment

The purpose of the thermal treatment test was to provide varying thermal conditions within a controlled environment to test the influence of environmental temperature on the stability of fs laser-induced modifications. The experimental procedure involved keeping control and structured glass at a particular temperature (ranging from −40 °C to +85 °C) for a period of 10 min per step, for 200 cycles. Between the cycles, the samples were left to cool down for 30 min at +25 °C. The equipment for this durability test involved a laboratory refrigerator (Electrolux, Stockholm, Sweden), which was set at +4 °C and −17.5 °C for the refrigerating and the freezing compartments, respectively. In order to achieve a temperature of −40 °C, a commercially available freezing spray (Motip freezer, Wolvega, Holland) was used to spray over the samples every 2 min for the 10 min step. The temperature was monitored by a non-contact thermometer (digital thermometer TP101, Handson technology, Masai, Malaysia). To reach temperatures of +25 °C and above, a drying oven with controlled temperature (Nahita 631 PLUS, AUXILAB S.L., Navarra, Spain) was utilized.

#### 2.5.2. Simulation of Daylight Effects via Exposition of Materials to UV Light

For the simulation of daylight effects, the samples were irradiated with UV light in a controlled environment (based on ISO 4892-3) [22,23]. Fs laser-treated and control samples were placed under a commercially available UV light (miniWH, China) emitting within the 390–400 nm range. The desired temperature settings were achieved via Humidity Oven (Nahita 631 PLUS, AUXILAB S.L., Spain). The temperature was set at 60 °C, and humidity was set at 50% for a period of 1000 h.

#### 2.5.3. Evaluation of Physical Endurance of Samples by Abrasion Test

The surfaces of the glass samples were subjected to an abrasion test by a Taber Abraser (Teledyne Taber, Erichsen, Germany—standard abrasion tester, model 503, patent N 2287148) to monitor the changes in morphology and optical properties after a physical endurance test. The test was performed with a CS-10-type abrading wheel, with a load of 50 g for 200 cycles.

#### 2.5.4. Evaluating the Chemical Resistance of the Samples

The surfaces of both laser-processed and control glass samples were tested for chemical resistance in acidic and neutral conditions. The methodology for this test involved the immersion of samples first in HCl (0.5 M) and then in NaCl solution (5%) for a period of 48 and 72 h, respectively. The samples were fully immersed in an enclosed vessel and kept under constant temperature for the chosen period of time at 40 °C ± 2 °C for the acidic solution and 35 °C ± 2 °C for the salt solution. After samples were taken out, they were washed under running distilled water and initially inspected for a change in the overall appearance, including color change, transparency, cracks, blistering, and other physical defects.

#### 2.5.5. Monitoring Degree of Dust Retention

The purpose of the dust retention test was to compare the degree of dust retention between fs laser-structured and nonstructured glass surfaces. Environmental dust particles were spread over the surfaces upon which the samples were kept at 80 °C for 2 h. After dusting the samples, their surfaces were cleaned following the described steps. The surfaces were evenly exposed to compressed air at 3 bar for 30 s. Then the samples were immersed in distilled water for 2 min under mild stirring, after which they were rinsed under running water. The final step involved the drying of the samples overnight at room temperature. Transmittance was measured before and after the test.

## 3. Results

### 3.1. Characterization of Laser-Processed and Unprocessed Samples Before Durability Test

#### 3.1.1. Morphological Analysis of fs Laser-Structured Glass Surfaces

**Figure 2:**
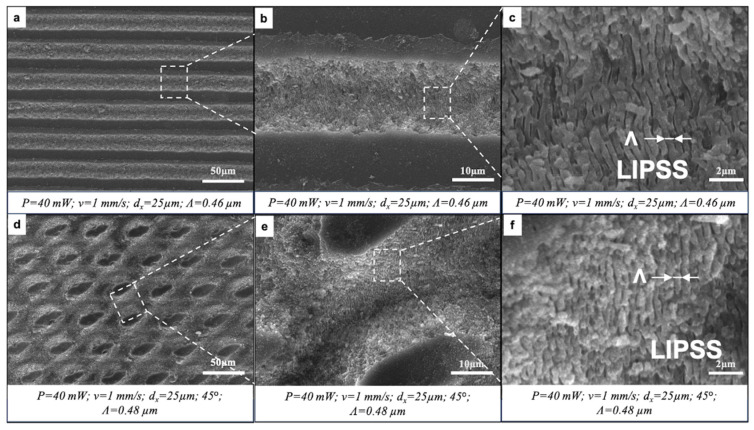
Formation of parallel and crossed microchannels bearing LIPSSs by fs laser irradiation. (**a**) P = 40 mW; v = 1 mm/s; d_x_ = 25 μm. (**b**,**c**) Magnified regions of (**a**), indicated by dashed boxes and lines. (**d**) P = 40 mW; v = 1 mm/s; d_x_ = 25 μm; 45° of intersection between two patterns. (**e**,**f**) Magnified regions of (**d**), indicated by dashed boxes and lines.

**Figure 3:**
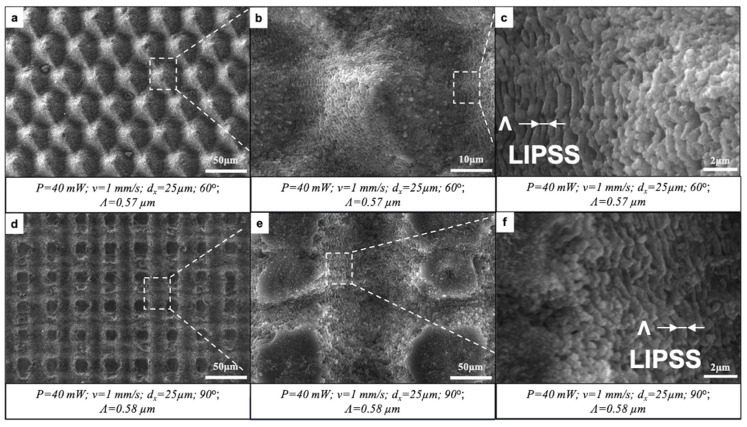
Formation of parallel and crossed microchannels bearing LIPSSs by fs laser irradiation. (**a**) P = 40 mW; v = 1 mm/s; d_x_ = 25 μm; 60° of intersection between two patterns. (**b**,**c**) Magnified regions of (**a**), indicated by dashed boxes and lines. (**d**) P = 40 mW; v = 1 mm/s; d_x_ = 25 μm; 90° of intersection between two patterns. (**e**,**f**) Magnified regions of (**d**), indicated by dashed boxes and lines.

Glass samples were laser-processed by applying the following working parameters: P = 40 mW; v = 0.5 ÷ 2 mm/s; d_x_ = 25 μm; and either a single layer of laser-inscribed lines or two or three layers intersecting at 45°, 60° or 90°. The initial observation of fs laser-induced surface patterns was performed using SEM. The results showed the formation of clearly defined parallel or intersecting microchannels of approximately 18 μm in width (Figure 2 and Figure 3). The crossing of patterns at a certain angle created sharp cones (60°) or domes with flat, unprocessed tops (45°, 90°). No heat-affected zones with collateral thermal damage were noted, confirming the precision of fs laser processing. The fs laser–matter interaction led to the appearance of laser-induced periodic surface structures (LIPSSs) with a period close to the wavelength of the fs laser (800 nm). The LIPSS period (Λ) was defined from the SEM images by obtaining multiple period measurements from diverse grooves regions, and afterwards, the average value was taken. The microchannels intersecting at a certain degree formed slopes at the intersections (Figure 3b,c,f) onto which the LIPSSs preserved their morphology and periodicity. The orientation of the LIPSSs was orthogonal to the direction of the laser beam raster scan.

**Figure 4:**
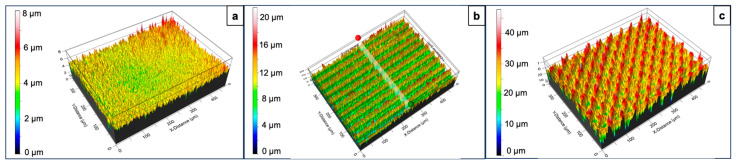
Surface profiles of control and laser-processed glass surfaces obtained by optical profilometry. (**a**) Control surface; (**b**) P = 40 mW; v = 1 mm/s; d_x_ = 25 μm; (**c**) P = 40 mW; v = 1 mm/s; d_x_ = 25 μm; 45° of intersection between two patterns.

**Figure 5:**
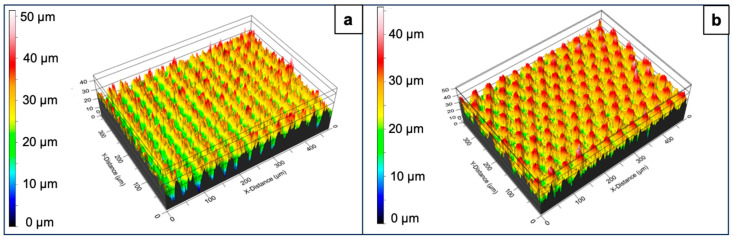
Surface profiles of control and laser-processed glass surfaces obtained by optical profilometry. (**a**) P = 40 mW; v = 1 mm/s; d_x_ = 25 μm; 90° of intersection between two patterns; (**b**) P = 40 mW; v = 1 mm/s; d_x_ = 25 μm; 60° of intersection between two patterns.

The surfaces of the laser-processed samples were further characterized by an optical profilometer, which provided more detailed information on the spatial features of the patterns (Figure 4 and Figure 5). The average depth of the parallel microchannels was 7.32 μm. This depth is expected to increase with the number of inscribed patterns. The motifs intersecting at 45°, 60°, and 90° showed average depths of 19.9 μm, 17.02 μm and 21.02 μm, respectively. Another important parameter monitored with the change in the applied laser parameters was surface roughness. In this case, the arithmetic mean height (Sa) was taken into consideration as it shows the average difference in height between points in a particular 3D surface when compared to a central mean plane. The Sa for the control untreated surface was 0.36 μm. Following the trend of the increasing depth of the microchannels, the Sa value for the surfaces with parallel patterns was 2.29 μm, while for the remaining surfaces, the parameter ranged between 4.3 and 4.7 μm.

#### 3.1.2. Physicochemical Characterization of fs Laser-Structured Glass Surfaces

The chemical composition of the soda–lime glass before and after laser structuring was investigated by means of energy-dispersive X-ray spectroscopy (EDX) (Table 1). The results indicated that the atomic percentage of oxygen is increasing with the number of inscribed patterns, which is most probably arising from the expulsion and rearrangement of material during the ablation process and could lead to the formation of surface oxides [24]. Furthermore, a decreasing trend was observed in the percentage of silicon. This could be explained by the fs laser-driven ion rearrangement during surface processing, which can affect the ratio between Si and O [24,25].

**Figure 6:**
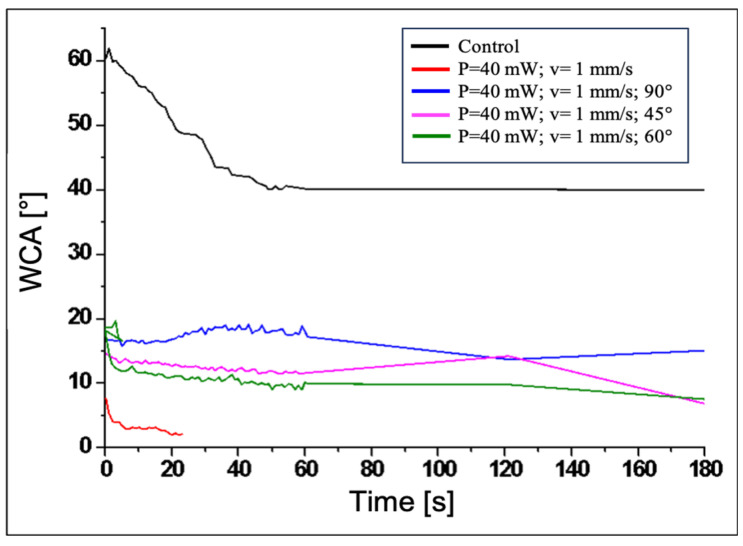
The evolution of the water contact angle on control and fs laser-structured surfaces. The angles (90°, 45°, 60°) indicate the angle of intersecting patterns.

As mentioned in Section 1, Introduction, superhydrophilicity and superhydrophobicity are key factors for developing a self-cleaning surface. Thus, the present study investigated the degree of surface wetting of the laser-processed glass substrates. In order to analyze how fs laser treatment affects the degree of wettability of glass surfaces, the water contact angle was measured on all types of surfaces. The graph in Figure 6 illustrates the contact angle evolution for the respective samples for a period of 3 min. The results revealed a 2.5-fold decrease in the mean values of the contact angles of the fs surface-treated samples compared to the control ones (Table 2). This observation indicated that laser structuring led to a strong increase in hydrophilicity. Furthermore, the samples bearing only parallel LIPSS-covered microchannels showed a superhydrophilic character, as 20 s after the initial drop–surface contact, complete surface wetting was observed, and measuring the angle was not possible. The reason for the higher wettability values for patterned surfaces was the laser-induced motifs which facilitated the spread of the water droplets according to the Cassie–Baxter models of rough surface wetting [26]. Figure 6 shows a slight increase in the contact angle towards the 2nd minute on surfaces with 45° of intersection, followed by a decline at the 3rd minute. This could be an example of how the droplet would spread upon the rising flat domes of the patterns, forming air pockets in between itself and the bottoms of the microchannels. Thus, an initial higher contact angle would be measured until the droplet spreads further along the motifs, resulting in lower angle values. Obtaining a superhydrophilic surface would mean that parallel LIPSSs lead to the complete distribution of the droplet over the surface. The enhanced hydrophilicity can be attributed to a transition toward the Wenzel wetting regime, where increased roughness amplifies the intrinsic wettability of the oxidized glass surface. Additionally, LIPSSs introduce nanoscale capillary channels that facilitate rapid liquid spreading. This could potentially be a factor for altering the self-cleaning properties of the structured glass layer on PV systems, as incoming water could wash off residual particles on the surface.

#### 3.1.3. Investigating Effects of fs Laser Structuring on Optical Properties of Glass

**Figure 7:**
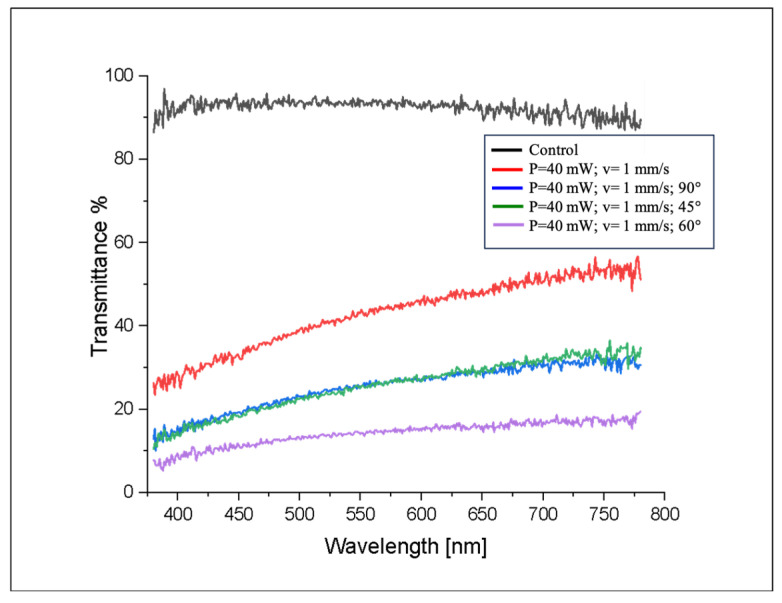
Transmittance [%] of control and fs laser-modified glass surfaces.

In order to realize self-cleaning surfaces for PVs, it is of key importance that the materials used have excellent optical properties. The results from the transmittance studies carried out in the spectral range between 380 and 780 nm on control and laser-treated glass samples are summarized in Figure 7. As expected, the untreated control samples exhibited the highest degree of transmittance as the respective percentages remained between 80 and 100% for the entire spectrum range. All fs laser-structured surfaces had increased transmittance toward longer (red) wavelengths compared to shorter (UV/blue) ones, with transmittance rising monotonically from ~0–20% at 380 nm to 50–90% at 780 nm. Fs laser texturing induces micro- and nanoscale structures, which act as diffraction gratings, reducing transmittance—particularly at short wavelengths—though increasing reflection and scattering. The samples with parallel microchannels exhibited higher transmittance than the other laser-structured samples, as their structures preserved grating coherence, potentially enabling antireflective benefits at selected wavelengths.

The samples processed with three crossed patterns transmitted the lowest amount of light (<20% across the spectrum), since multiple processing layers led to the highest amount of ablation and redeposition of material, creating a more opaque surface. There exists a trade-off between enhanced hydrophilicity and optical transmittance. More complex hierarchical structures increase scattering losses due to enhanced light diffraction and multiple reflections within the micro- and nanostructures [13]. A clear trade-off between wettability and optical performance is observed. While the untreated glass exhibits high transmittance (80–100%), laser-structured surfaces show a reduced slight change in the transmission depending on structural complexity. Parallel microchannels retain moderate transmittance (up to ~50% at longer wavelengths), whereas intersecting hierarchical structures exhibit significantly lower values (10–40%) but demonstrate the strongest hydrophilic behavior. This highlights that increased surface roughness and hierarchy enhance wetting performance at the expense of optical efficiency due to increased light scattering. In contrast, the least processed surfaces reached 50% transmittance at longer wavelengths. These surface modifications primarily affect scattering without altering the intrinsic glass transmission band up to 1100 nm, making the material suitable for photovoltaic glass, which prioritizes high transmittance from the 350 to 1100 nm regions [27,28,29].

### 3.2. Characterization of fs Laser-Processed and Control Surfaces After Physicochemical Durability Tests

The samples chosen to undergo the series of durability tests, described in Section 2, Materials and Methods, were only control surfaces and ones comprising either parallel microchannels governed with LIPSSs or LIPSS patterns intersecting at 60° (P = 40 mW; v = 1 mm/s). Surfaces with the respective type of fs laser-induced modification were prepared and exposed to each of the durability tests. However, a separate group comprising controls, surfaces with parallel microchannels, and surfaces with microchannels intersecting at 60° was subsequently tested in all of the above-mentioned physicochemical conditions/durability tests.

#### 3.2.1. Morphological Characterization

**Figure 8:**
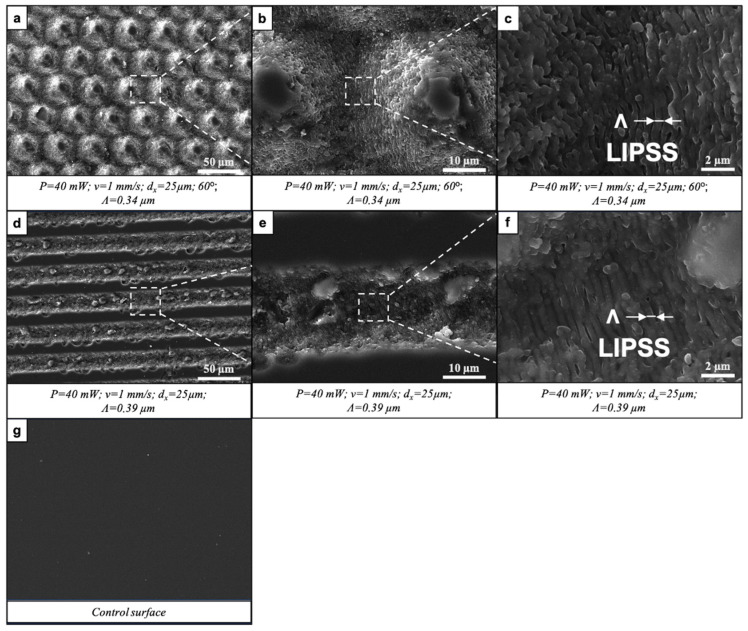
Fs laser-processed glass surfaces preserving their morphology after undergoing subsequent physicochemical durability tests. (**a**–**c**) P = 40 mW; v = 1 mm/s; d_x_ = 25 μm; 60° of intersection between three patterns. (**b**,**c**) Magnified regions of (**a**), indicated by dashed boxes and lines. (**d**–**f**) P = 40 mW; v = 1 mm/s; d_x_ = 25 μm. (**e**,**f**) Magnified regions of (**d**), indicated by dashed boxes and lines. (**g**) Control surface.

The initial morphological observation of the samples that underwent all durability tests subsequently revealed that the fs laser-induced surface modifications were preserved with slight changes. Figure 8 depicts SEM images of the three types of surfaces. In this figure, it can be seen that the peaks formed at the intersection of the patterns had flattened tops with an overall loss of sharpness of the patterns (Figure 8a,b). The LIPSSs, both on the slopes and within the parallel microchannels, also appeared smoothed with the partial fusing of individual ripples. Nevertheless, the overall micro- (channels, grade of grooves) and nano- (LIPSS) morphology was preserved, which shows the stability of the structures created and their potential for future application in creating self-cleaning PV panels.

**Figure 9:**
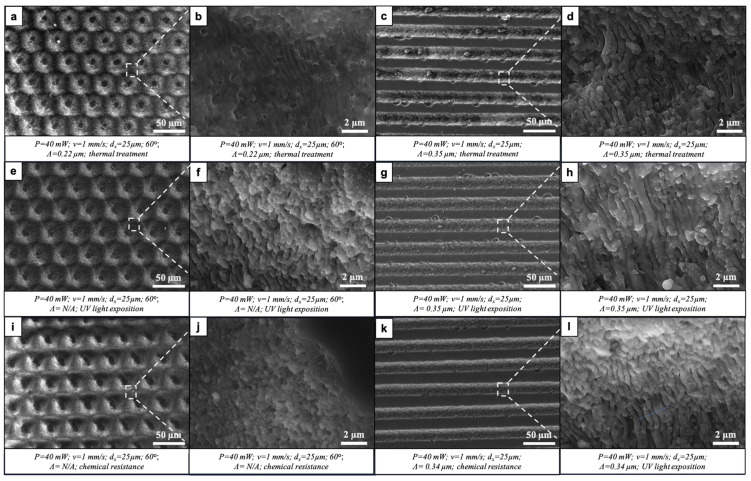
Morphology of fs laser-structured glass surfaces after being tested for physicochemical durability. (**a**–**d**) Surfaces designed based on intersecting and parallel modifications after undergoing thermal treatment. (**b**,**d**) Magnified regions of (**a**) and (**c**), respectively, indicated by dashed lines and boxes. (**e**–**h**) Surfaces after being exposed to UV light. (**f**,**h**) Magnified regions of (**e**) and (**g**), respectively, indicated by dashed lines and boxes. (**i**–**l**) Surfaces after tests on chemical resistance. (**j**,**l**) Magnified regions of (**i**) and (**k**), respectively, indicated by dashed lines and boxes.

**Figure 10:**
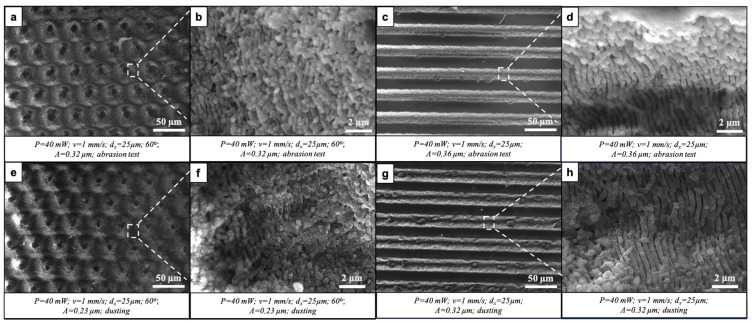
Morphology of fs laser-structured glass surfaces after being tested for physicochemical durability: (**a**–**d**) Surfaces bearing intersecting and parallel modifications after abrasion test. (**b**,**d**) Magnified regions of (**a**) and (**c**), respectively, indicated by dashed lines and boxes. (**e**–**h**) Surfaces bearing intersecting and parallel modifications after being dusted. (**f**,**h**) Magnified regions of (**e**) and (**g**), respectively, indicated by dashed lines and boxes.

Similar results were also noted when comparing surfaces before and after individual durability tests. Figure 9 presents SEM images of individual laser-modified surfaces after each of the tests. The overall conclusion of the morphological analysis was that both of the laser-induced patterns were preserved after each of the aforementioned tests. The LIPSS nanostructures, both in the parallel microchannels and on the slopes of the intersecting channels, had retained their morphology. Partial fusion and the loss of order of the nanostructures could be seen after the tests for thermal stability and chemical resistance (Figure 9f,j). Panels a–d in Figure 10 depict laser-structured surfaces after the abrasion test. The stronger smoothening of the peaks (Figure 10a) was expected; however, the general hierarchical micro- and nano-laser-induced morphology was preserved. The next step in our studies will focus on the quantitative analysis of morphological evolution, including the statistical evaluation of LIPSS periodicity, feature height, and surface roughness (Sa) before and after durability testing.

#### 3.2.2. Evaluation of Surface Wettability

**Figure 11:**
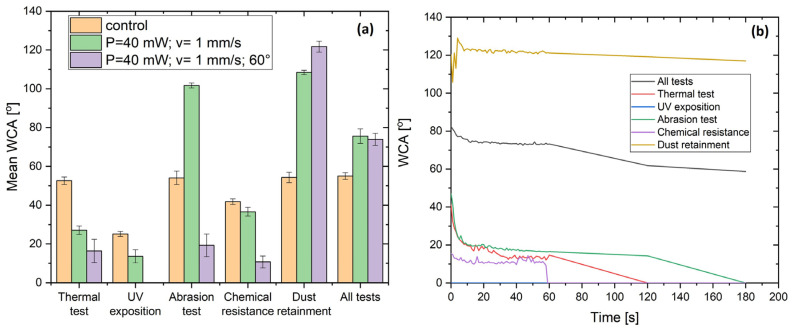
(**a**) Mean and (**b**) dynamic WCA values of fs laser-structured surfaces after durability tests.

Surface wettability was evaluated for both the samples that sequentially went through all durability tests and the ones that underwent individual tests. The mean values of the contact angles of all surfaces with the corresponding durability test are presented in the graph in Figure 11a. Overall, the results showed that the fs laser-structured surfaces remained more hydrophilic compared to the control samples. This trend was not followed only in the samples that were used for dust retention tests. In this case, surfaces with both types of modifications showed strong hydrophobicity with mean contact angles reaching 120°. Another case of stronger hydrophobicity (101.7°) was noted for the glass surface bearing parallel microchannels with LIPSSs that underwent the abrasion test. The observed transitions between hydrophilic and hydrophobic states can be attributed to changes in surface chemistry and adsorption processes. In particular, the accumulation of airborne hydrocarbons or residual contaminants within the micro- and nanostructures may reduce surface free energy, leading to increased hydrophobicity. Conversely, UV exposure and cleaning steps can promote surface reactivation through oxidation and hydroxylation, restoring hydrophilic behavior [15,30]. On the opposite side, the surfaces bearing microchannels intersecting at 60° appeared to be hydrophilic after exposure to UV light according to the described procedure (contact angle could not be measured, due to complete wetting of the surface). Apart from the dusting test, this laser-induced modification retained its hydrophilicity across the different durability tests with a mean contact angle of less than 20°, demonstrating a preserved hydrophilic surface. According to the surfaces whose wettability was analyzed after passing through all the tests, similar but slightly higher hydrophobicity compared to the controls was demonstrated.

Considering the average contact angle measurements, a closer look at the wettability of the surfaces with crossing patterns over the 3 min interval showed that by the end of the third minute, the surfaces had become fully wetted, leaving no measurable contact angle (Figure 11b). Overall, the findings of the wettability measurements confirmed that the glass surfaces structured with microchannels intersecting at 60° preserved their strong hydrophilicity after each of the different treatments, which confirms that the durability tests did not alter the physicochemical properties of the laser-induced structures.

#### 3.2.3. Analysis of Optical Properties of Laser-Structured Glass Surfaces After Durability Tests

**Figure 12:**
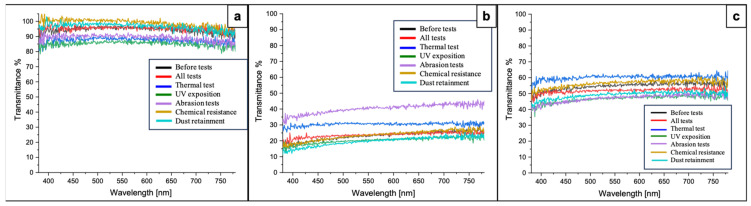
Transmittance [%] of control and laser-structured surfaces after durability tests. (**a**) Control surfaces; (**b**) P = 40 mW; v = 1 mm/s; d_x_ = 25 μm; 60° of intersection between three patterns; (**c**) P = 40 mW; v = 1 mm/s; d_x_ = 25 μm.

The transmittance of laser-processed and control surfaces after durability tests was measured and is presented in Figure 12. The results of the analysis showed that the control surfaces exhibited transmittance between 80% and 100% after all durability tests—Figure 12a. The surfaces with intersecting microchannels had the lowest transmittance, ranging between 10% and 40% for samples that underwent abrasion tests, showing the highest percentage among the rest—Figure 12b. The samples bearing parallel microchannels showed slightly higher transmittance—Figure 12c. When comparing the results from Figure 7 and Figure 12, it can be highlighted that the overall transmittance [%] remained the same or increased for laser-structured surfaces after being subjected to the series of durability tests. Thus, the different treatments did not affect the optical properties of the surfaces, contributing to the evidence that fs laser-induced patterns exhibit good durability and can withstand varying physicochemical conditions, with a minimal decline in their overall qualities.

## 4. Discussion

The present study aimed at proposing an experimental methodology for overcoming the practical limitations of photovoltaic systems. The methodology is based on the fs laser structuring of glass surfaces for obtaining hierarchical micro- and nanostructures that would contribute to the development of a self-cleaning surface. This study involved the fs laser processing of glass substrates and subsequent durability tests, simulating real-life conditions, after which their morphological appearance, wettability and optical properties were evaluated.

The performed evaluation of chemical durability is very important in the case of the long-term outdoor usage of laser-structured glass surfaces in photovoltaic panels. In the current research, the chemical tests performed by immersion in acidic (HCl, 0.5 M) and saline (NaCl, 5%) environments were performed with the aim of simulating aggressive environmental conditions, for example, marine atmospheres or acidic rain environments. The obtained results demonstrated that the femtosecond laser-induced micro/nanostructures remained unchanged. And only very few changes in LIPSSs expressed in smoothing were observed. Additionally, deterioration in WCA or optical performance was not detected. In summary, the demonstrated chemical stability, in combination with the absence of additional chemical coating-related degradation mechanisms, shows that femtosecond laser processing could be an option for achieving sustainable glass surfaces with tunable wettability, for long-term exploitation in solar panels. Future experimental work is needed to extend outdoor field testing to further validate long-term chemical durability.

Biological fouling is one of the main concerns for the long-term outdoor application of laser-processed glass surfaces for solar panel applications. It was demonstrated in the current study that fs laser-textured surfaces possess highly hydrophilic properties. These specific conditions could suppress the microorganism attachment. The formation of a continuous water film can govern the removal of biological remnants during rainfall. In contrast to coatings based on polymers, laser-created structures in the form of LIPSSs and hierarchical ones would limit the formation of the organic thin film, thus leading to the diminishing of potential nutrient sources for bacterial development. This in turn would hinder the formation of biofilm and also increase the risk of coating delamination.

Femtosecond laser-patterned glass proved highly robust under the simulated environmental tests. For example, the proposed thermal cycling protocol (−40–85 °C for 200 cycles) closely matches the IEC 61215 standard for PV modules, and the fs laser micro/nanostructures remained intact with no collateral thermal damage [31]. Likewise, after 1000 h of UV irradiation (60 °C, 50% RH), chemical resistance and abrasion, the hierarchically laser-textured patterns were still clearly visible and preserved. In practical terms, this means that the engineered microchannels and LIPSSs remain without chipping or collapse, so the surface topography—and thus its wetting behavior—was maintained. This endurance is consistent with other reports on designed ultrafast laser surfaces that retain their morphology over time [32,33,34]. Moreover, optical transmittance did not deteriorate; in fact, the structured surfaces showed the same or slightly higher visible transmittance after testing. Overall, the proposed fs laser processing methodology yields durable surfaces with a potential self-cleaning effect that withstand PV module stress tests without losing their designed features.

Fs laser-textured samples remained more hydrophilic than the unprocessed controls throughout durability testing. Surfaces patterned with intersecting microchannels (crossing at 60°) exhibited strong hydrophilicity: water droplets spread completely within three minutes after initial droplet–surface interaction, and contact angles stayed near zero, even after thermal, UV (full wetting upon initial contact), abrasion, and chemical aging. Similar results were found in the research of Ahmad et al., who reported that uncoated laser-structured steel meshes achieved lower contact angles (≈41°) and that adding a nanocoating further reduced the hydrophilic behavior (≈14°), which was preserved for a 30-day period [32]. In our case, the laser-induced hierarchical patterns on glass substrates (without further coating) similarly led to the rapid spreading of water, and this behavior proved stable after the durability tests. However, an important observation from the present study is that dust deposition can substantially alter the wetting behavior of the laser-structured surfaces as the patterned substrates became highly hydrophobic (water contact angles ≈120°). Specifically, the adsorption of residual organic and inorganic particles at the micro- and nanoscales can induce a transition from hydrophilic to hydrophobic behavior. This dynamic wettability response is likely to occur under real environmental conditions and may play a beneficial role in self-cleaning processes by facilitating the detachment and removal of accumulated contaminants. Laser-textured oxide surfaces are known to readily adsorb airborne hydrocarbons within nanoscale features, which lowers the surface free energy and induces a hydrophilic-to-hydrophobic transition after environmental exposure, as reported by Fürbacher et al. [35]. Nevertheless, intersecting patterns performed better by means of their hydrophilic behavior: their deep channels and slopes led to the strongest hydrophilicity even after performing all durability tests, whereas simple parallel channels were hydrophilic but to a lesser extent. This trade-off matches prior observations that increasing micro/nano-complexity boosts wetting control [32,36]. The wettability behavior of fs laser-structured surfaces is governed by a combination of surface morphology and chemical composition. After laser processing, the formation of oxide layers and hydroxyl groups leads to high surface energy and high hydrophilicity. The adsorption of dust and environmental contaminants within the hierarchical micro- and nanostructures reduces the surface free energy, resulting in hydrophobic recovery. Such bidirectional wettability transitions have been widely reported for laser-textured materials and are strongly influenced by environmental exposure conditions [37,38]. From an application perspective, both hydrophilic and hydrophobic states can contribute to self-cleaning behavior through different mechanisms; however, controlling the long-term stability of the wetting state remains a key challenge [39]. Additionally, increasing structural complexity enhances wettability but introduces higher optical scattering losses, highlighting the need to optimize the balance between surface functionality and optical performance.

Importantly for solar applications, the fs laser textures did not irreversibly reduce transmittance. Although the transmittance measurements in this study were limited to the visible spectral range (380–780 nm) due to instrumental constraints, previous studies have shown that femtosecond laser-induced surface structuring predominantly influences scattering at shorter wavelengths. The intrinsic transmission band of soda–lime glass extends into the near-infrared region (up to ~1100 nm), which is highly relevant for photovoltaic applications and is generally not significantly altered by such surface modifications [29]. Before durability testing, the surfaces with parallel microchannels transmitted between ~30 and 50% of visible light, whereas triple-crosshatch patterns did not reach more than 20%. After the durability tests, the overall transmittance was essentially unchanged or even slightly improved, showing that no new scattering centers (like microcracks or residues) formed. This aligns with the results of other studies of self-cleaning coatings, which observed a modest transparence loss in exchange for cleaning function. For example, Shirazi et al. found that adding a self-cleaning carnauba wax layer reduced transmittance on perovskite solar cells, but nonetheless, it kept the surfaces superhydrophobic and preserved their performance [36].

The proposed eco-friendly methodology in this paper demonstrated that pure laser-induced patterns on glass have the potential to deliver sustained wettability control, without the need for additional functionalization. A similar study design but on stainless steel and not on glass was proposed by Fürbacher et al., in which it was noted that fs laser-structured stainless steel loses its superhydrophobic properties after 100 h of UV light exposure [35]. In contrast, our results showed that the intersecting laser-induced patterns became superhydrophilic after 1000 h of UV exposure. Based on the findings from the literature and comparing them to the results of the current study, it could be stated that the design of the patterns’ geometry could offer an adjustment in surface properties. More complex 3D motifs (microchannel intersecting at 60°) maximize hydrophilicity; however, they reduce light transmittance. On the other hand, a simpler motif (single parallel microchannels) either preserves or slightly reduces transmittance but compromises on hydrophilicity.

These findings suggest that laser-processed surfaces possess good resistance to chemical treatments under conditions simulating outdoor exploitation. Moreover, unlike conventional self-cleaning coating approaches, their functionality is preserved on glass surfaces mainly through morphological modification. This decreases the common failure mechanisms related to delamination appearance or chemical coating dissolution.

Future optimization should focus on reducing structure depth and density, as well as exploring hybrid surface designs that preserve antireflective behavior while maintaining favorable wetting properties. Although femtosecond laser processing offers high precision and enables chemical-free surface functionalization, its scalability remains a challenge due to the relatively low throughput associated with serial processing. Nevertheless, recent advances in high-repetition-rate laser systems, multi-beam and parallel processing strategies, and high-speed galvanometric scanning have significantly improved processing efficiency. Compared to conventional coating-based approaches, such as the spray-coating of a TiO_2_–SiO_2_–PAA superhydrophilic layer or silica/sol–gel coatings, laser structuring eliminates issues related to coating adhesion, degradation, and environmental impact, offering a potentially more durable long-term solution despite higher initial processing costs [40,41,42].

In summary, our study provides new evidence that intersecting fs laser textures display a combination of high durability, strong hydrophilicity, and acceptable optical properties—a combination not widely reported before—thus representing a robust methodology for creating PV anti-soiling surfaces.

## 5. Conclusions

In this work, we demonstrated that femtosecond laser texturing can be used to reliably tailor the wetting and optical behavior of glass surfaces relevant to photovoltaic applications. By generating controlled micro- and nanoscale motifs—either parallel channels or intersecting patterns—the surfaces acquired distinct wetting characteristics that remained largely unchanged even after exposure to a comprehensive set of durability tests. Thermal cycling, prolonged UV irradiation, chemical exposure, and mechanical abrasion caused only minor smoothing of the textured features, while the overall architecture of the laser-induced structures was preserved. This stability was reflected in wettability measurements: surfaces patterned with intersecting microchannels, in particular, maintained their strong hydrophilic response and, in several cases, showed complete wetting within minutes.

Importantly, the optical performance of the structured surfaces did not degrade as a result of durability testing. The transmission values remained comparable to, or slightly higher than, those measured before environmental exposure, indicating that laser processing did not trigger additional scattering or structural damage. Taken together, the results show that the proposed laser processing approach can generate robust topographies without relying on chemical coatings or complex post-processing steps. The durability of the patterns, combined with their tunable wetting behavior, suggests that this method may offer a practical route toward long-lasting front surfaces for photovoltaic systems.

## Figures and Tables

**Table 1:** EDX analysis of control and fs laser-processed soda–lime glass surfaces.

Element	Atomic %		
	Control	P = 40 mW; v = 1 mm/s	P = 40 mW; v = 1 mm/s; d_x_ = 25 μm; 60° of Intersect.
C	1.51	2.01	3.98
O	61.3	63.75	65.28
Na	8.22	7.83	6.78
Mg	1.74	1.69	1.38
Al	0.75	0.63	0.55
Si	22.73	20.66	18.7
K	0.3	0.29	0.26
Ca	3.42	3.15	3.07

**Table 2:** Mean values of water contact angles of control and fs laser-structured glass surfaces.

Sample	Mean Contact Angle [°]
Control	43.31
P = 40 mW; v = 1 mm/s	5.1
P = 40 mW; v = 1 mm/s; d_x_ = 25 μm; 90° of intersect.	17.49
P = 40 mW; v = 1 mm/s; d_x_ = 25 μm; 45° of intersect.	12.37
P = 40 mW; v = 1 mm/s; d_x_ = 25 μm; 60° of intersect.	18.82

## Data Availability

The original contributions presented in this study are included in the article; further inquiries can be directed to the corresponding authors.

## Abbreviations

The following abbreviations are used in this manuscript:

LIPSS	Laser-induced periodic surface structure
UV	Ultraviolet
PV	Photovoltaic
SEM	Scanning electron microscopy
WCA	Water contact angle
